# The COVID-19 pandemic in the hotel industry: the application of hygiene and safety protocols by hotel employees

**DOI:** 10.1590/S1678-9946202668024

**Published:** 2026-03-13

**Authors:** Cláudia Regina Rodrigues Sarralheiro Butuhy, Expedito José de Albuquerque Luna

**Affiliations:** 1 Universidade de São Paulo, Faculdade de Medicina, Instituto de Medicina Tropical de São Paulo, Programa de Pós-Graduação em Medicina Tropical, São Paulo, São Paulo, Brazil; 2 Universidade de São Paulo, Faculdade de Medicina, Departamento de Medicina Preventiva, São Paulo, São Paulo, Brazil; 3 Universidade de São Paulo, Faculdade de Medicina, Instituto de Medicina Tropical de São Paulo, São Paulo, São Paulo, Brazil

**Keywords:** Pandemic, COVID-19, Travel medicine, Hygiene protocol, Hotels, Employees, Brazil

## Abstract

This study sought to understand how the application of hygiene and safety protocols by hotel companies in Sao Paulo city, Brazil, occurred during the COVID-19 pandemic. The first objective of the study was to find, based on the information provided by employees who worked in certain hotels via questionnaires from March 2020 to December 2022, which protocols were applied in the studied period and which measures were definitively incorporated into the hygiene schemes of these hotels. Secondly, this study aimed to assess how the interaction and adaptations to the implemented hygiene protocols took place to find the measures that have become hygiene habits in everyday life. The literature on the applied health protocols during the pandemic was reviewed. A cross-sectional descriptive study was conducted with 421 respondents. This study found a significant association between sex and the adoption of protective measures during the pandemic. Women adhered more to protective measures than men, and this association remained after the pandemic. Employees who worked in the food and beverage area contracted COVID-19 more often than employees in other hotel areas. Those with higher education levels received more doses of COVID-19 vaccines than those with lower levels of education. Finally, this study found a direct relationship between the quality standard of the hotels and the frequency of training received by employees and a high adherence to vaccination against COVID-19 that was lower at higher educational levels.

## INTRODUCTION

The main difficulty for the healthcare providers servicing travelers is that patients rarely consult before traveling since they lack knowledge about the risks in their destination. Adopting preventive measures, lending advice to travelers before their trip, and providing them with follow-up after their return could minimize a considerable part of these risks. A 2018 survey showed a high proportion of occurrence of health problems during trips in participants.

The COVID-19 pandemic showed the fragility of this process, directly affecting national tourism. According to data released in 2020 Brazil had 10,501 hotels and flats, totaling 556,180 rooms for 2019. That survey estimated that Sao Paulo city had about 410 hotels and around 46,000 rooms^1^. However, in the largest virtual platform for booking rooms in lodging facilities currently in operation in the Brazilian territory, Sao Paulo city, in Sao Paulo State, has 1.241 lodging facilities classified as hotels and approximately 50,000 rooms^2^.

The application by hotel companies of hygiene and safety protocols during the COVID-19 pandemic (suggested by global health organizations, advocated by national authorities, or those developed by the companies themselves) showed that the means of accommodation sought to adapt to the requirements of protecting their guests and employees against COVID-19 and to ensure its operation within the rules in force at the time. In 2019^3^, the economic hotel industry in Sao Paulo city ended with an average occupancy of its housing units in the order of 59.49%. However, with the COVID-19 pandemic, in the following year, at the height of the crisis in the hotel market (from March to October 2020), 91% of hotels closed temporarily or permanently, with their occupancy rates decreasing to record lowest percentages (close to 10%).

In this scenario, regarding the safety in the work environment and to regain the trust of guests and employees and comply with mandatory government standards, hygiene and safety protocols were implemented. These protocols became essential factors to disseminate concepts and attitudes of protection against the contamination of environments and the spread of the virus during the pandemic^4,5^.

Despite the automation of several processes in the hotel industry related to guest service, many still take place manually. Examples include check-in and check-out services, room cleaning, room service, laundry dispatch, meals, among others, forcing the industry to take greater care with health emergencies, such as the one we have recently experienced.

As this study found no research on the use of hygiene protocols in hotel companies, the result of this study can support the health and hotel fields on the positive and negative aspects of the applied protocols and guide hotel companies in the Sao Paulo city (in addition to Brazilian tourism and health agencies) to better inform them about the taken protocols and measures to prevent the transmission of COVID-19.

The main objective of this study was to find, via questionnaires answered by employees of hotels in the Sao Paulo city, how the interaction and adaptations to the hygiene protocols implemented by hotel companies occurred. Moreover, we sought to know what protocols hotels applied during the pandemic, if employees knew the applied protocols in the hotel in which they worked during the pandemic, which measures have been definitively incorporated into the daily life of hotels, and to know which measures have become daily habits after the pandemic.

## MATERIALS AND METHODS

The literature on the hygiene and safety protocols created for hotels during the COVID-19 pandemic^6-11^ was retrieved by the string "Hotel," "Tourist," and "COVID-19" and reviewed. The main sources of this review were the Brazilian Ministry of Tourism and the Ministry of Health, national and state hotel associations, independent hotels and hotel chains in Sao Paulo city, and the following scientific databases: VHL, Elsevier, Medline, PubMed, SciELO, and Scopus.

Considering the estimated proportion of 5% of one-star hotels, a 1% variability in this estimate, a 95% confidence level, and a total of 450 hotels, the sample size was calculated at 362 respondents. Adding 10% to compensate for possible losses, the final sample size was calculated at 400 respondents. A field survey was carried out with hotel professionals. Starting at each hotel with a seed respondent, in addition to answering the questionnaire, they were asked to introduce the study to their coworkers and professional network so that they could disseminate the survey so it would reach the desired number of respondents.

A semi-structured questionnaire with 22 questions was used. The initial 20 interviewees were used as a pre-test, a number considered satisfactory to assess the understanding of the questionnaire. The questionnaire was answered in person or electronically using a link that was sent to the e-mail of people who were willing to answer the questionnaires. The digital survey platform "Survey Monkey" was used for data collection and analysis.

The hotels were classified according to their self-declared standard, into one, two, three, four or five "stars." Based on this classification, the following numbers (approximate percentages) of rooms per category in the Sao Paulo city were obtained: one-star rooms represent 5%; two-star rooms, 10%; three-star rooms, 35%; four-star rooms, 30;% and five-star rooms, 20%. In the data analysis, the hotels were grouped into two clusters, the first including one- and two-star and unclassified hotels. The second cluster was composed of three-, four-, and five-star hotels.

The results are shown as frequency tables and graphs. The chi-squared test was used to test differences between proportions and the chi-squared test for linear trends was used for ordinal variables. The Student's *t*-test and analysis of variance were used to test the difference between means.

### Ethics

Pre-testing and the final questionnaire were carried out after the interviewees had agreed to it by signing an informed consent form. This study was approved by the ethics review board of our institution (statement Nº 5.687.569, October 6, 2022).

## RESULTS

Our literature review retrieved 978 articles by using the string "Hotel," "Tourist," and "COVID-19." After the analysis of their titles and abstracts, this study chose 43 articles on the impacts on the tourism market of the COVID-19 pandemic. The remaining 935 articles addressed topics such as post-pandemic hotel marketing, post-pandemic human resource management, financial projections, and future hotel statistics. They explained how these events occurred in countries such as the United Kingdom, China, France, and Australia (for example), how quarantine hotels and hospitalization hotels operated in Italy and Spain (lodging facilities converted into hospital settings to care for people with milder COVID-19 symptoms), and homeless hotels, used in the United States and some northern European countries to house people in vulnerable housing situations and to reduce their chances of becoming infected. Some other articles discussed hygiene and safety protocols as essential for the return of the confidence of employees and tourists in traveling again and the responsibilities of tourism companies in helping to control the spread of the pandemic^12-27^.

However, these studies evinced a gap as no chosen article directly researched the employees of these companies. This study applied its survey questionnaire from September 1, 2023, to March 4, 2024. Of the 421 respondents, 51.3% (216 people) were men and 48.7% (205 people) were women, 66.3% (279 people) aged from 21 to 30 years; 23.3% (98 people), from 31 to 40 years; 9.3% (39 people) from 41 to 50 years; and 1.2% (5 people), 51 years or above (Table 1).

**Table 1:** Participants’ characteristics

Variables		n	%
**Gender**			
	Male	205	48.7
	Female	216	51.3
**Age**			
	18 – 30	279	66.3
	31 – 40	98	23.3
	41 – 50	39	9.3
	51 or older	5	1.2
**Marital status**			
	Married	144	34.2
	Single	241	57.2
	Divorced	36	8.6
**Education**			
	Elementary	48	11.4
	High school	77	18.3
	Undergraduate	265	62.9
	Graduate studies	31	7.4
**Have you had COVID-19?**			
	Yes	229	54.4
	No	192	45.6
**How many times have you had COVID-19?**			
	1 time	138	32.8
	2 times	59	14.0
	3 times	32	7.6
**Have you been vaccinated against COVID-19?**			
	Yes	405	96.2
	No	16	3.8
**How many doses of the vaccine have you taken?**			
	1 dose	29	6.9
	2 doses	33	7.8
	3 doses	157	37.3
	4 doses	55	13.1
	5 doses	131	31.1
**Department of work**			
	Lodging	274	65.1
	Food and beverage	90	21.4
	Management/Financial	57	13.5
**Hotel classification**			
	Non-classified	32	7.6
	1 star	63	15.0
	2 stars	67	15.9
	3 stars	83	19.7
	4 stars	103	24.5
	5 stars	73	17.3
**Frequency of training on the use of hygiene and safety protocols**			
	None	22	5.2
	Once	147	34.9
	Quarterly	14	3.3
	Bimonthly	16	3.8
	Monthly	94	22.3
	Biweekly	25	5.9
	Weekly	103	24.5

Regarding interviewees’ education, 29,7% had complete high school, 62.9% (265 people) had an undergraduate degree, and 7.4% (31 people) had a graduate degree.

When asked if they had had COVID-19, 45.6 (192 people) answered "NO" and 54.4% (229 people) said "YES". When asked if they were vaccinated against COVID-19, 3.8% (16 people) said "NO" and 96.2% (405 people) said "YES". Of these 32.3% (131 people) had received five doses; 13.6% (55 people), four; 38.8% (157 people), three; 8.1% (33 people), two; and 7.2% (29 people), a single dose.

Regarding in which area or department they worked, 65.1% (274 people) belonged to a lodging department; 21.4% (90 people), to food and beverages; and 13.5% (57 people), to a administrative/financial/marketing department. Regarding the classification the hotels adopted, of the 421 respondents, 7.6% (32 people) stated that the hotel in which they worked "fit no classification;" 17.3% (73 people) reported working in a five-star hotel; 24.5 (103 people), in a four-star one; 19.7% (83 people), in a three-star one; 15.9% (67 people), in a two-star one; and 15.0% (63 people), in a one-star one.

Regarding the use of hygiene and safety protocols (HSP) by the hotel they worked, all stated "YES". Regarding training on "HSP": 94.8% (399 people) stated having received it and 5.2% (22 people) said otherwise. Regarding the frequency of training on how to apply the HSP, 34.9% (147 people) stated that they had received training only once, 24.5% (103 people) had received weekly training, 22.3% (94 people) reported monthly training, 5.9% (25 people) had received training biweekly, 3.8% (16 people) bimonthly, and 3.3% (14 people), quarterly.

The following constitute respondents’ most frequently marked answers to the question about which of the 52 items of the "HSP" the hotel in which they worked implemented: "use alcohol gel to disinfect your hands," "maintain physical distancing," "had dispenser of hand sanitizer in social areas," and "had dispenser of hand sanitizer in the internal areas" as all participants (100% - 421 people) marked these items. The least marked were the following: "establish an isolation area for confirmed cases of COVID-19 infection, with restriction on the movement of people and employees" with 3.8% (16 people); "working from home," with 8.6% (36 people), and "keeping one person to serve the other employees at the cafeteria buffet," with 15.0% (63 people) (Table 2).

**Table 2:** Hygiene and safety protocol items in use at the hotels during the COVID-19 pandemic (421 answers)

Variable	n	%
1. Wear masks correctly at work	396	94.1%
2. Use alcohol gel to disinfect hands	421	100.0%
3. Maintain physical distancing	421	100.0%
4. Have acrylic barriers at the reception	334	79.3%
5. Keep employees belonging to risk groups away from work	70	16.6%
6. Reduce the number of employees entering the rooms (cleaning and/or room service)	383	91.0%
7. Pack bed linen and towels	353	83.8%
8. Pack plates, glasses and cutlery	366	86.9%
9. Reduce the number of passengers in elevators	381	90.5%
10. Have mats with disinfectants	401	95.2%
11. Work from home	36	8.6%
12. Check the employees’ temperature upon arrival at work	317	75.3%
13. Remove self-service coffee and/or other drinks from social areas (thermal/machine)	334	79.3%
14. Have alcohol gel dispensers in the elevators	371	88.1%
15. Remove newspapers and magazines from the lobbies/reception	355	84.3%
16. Use soap and disposable towels (washbasins) in addition to activated trash cans without using their hands.	407	96.7%
17. Conduct campaigns, via online communication channels, about the hotel policy on safety protocols	409	97.1%
18. Reduce the amount of furniture in social areas	211	50.1%
19. Clean rooms with ventilated areas	371	88.1%
20. Perform wet cleaning instead of dry cleaning and use of a vacuum cleaner	347	82.4%
21. Make reservations for customers in restaurants and bars with reduced spaces	128	30.4%
22. Keep employees to serve guests at buffets, which must be completely closed	259	61.5%
23. Install acrylic/glass protection at the buffet in restaurants	226	53.7%
24. Do not perform table mise en place to avoid cross-contamination	243	57.7%
25. Provide and maintain good ventilation in social areas and back offices	197	46.8%
26. Disinfect leisure areas (gyms, saunas, solariums, etc.) after use and only with reservations	142	33.7%
27. Disinfect air conditioning weekly	389	92.4%
28. Cover payment machines with plastic film	408	96.9%
29. Encourage contactless payments	406	96.4%
30. Disinfect luggage upon check-in	278	66.0%
31. Cover TV and air conditioning remote controls with plastic film	342	81.2%
32. Have signs with COVID-19 prevention guidelines	408	96.9%
33. Disinfect TVs weekly	376	89.3%
34. Cover power cables with plastic film	105	24.9%
35. Use disposable amenities as a priority	367	87.2%
36. Disinfect room key cards (check-in)	402	95.5%
37. Remove trash more frequently	358	85.0%
38. Check guests’ temperature upon checking in at the hotel	124	29.5%
39. Disinfect the key card after the guest returns it (check-out)	383	91.0%
40. Have alcohol gel dispensers in social areas	421	100.0%
41. Have alcohol gel dispensers in internal areas	421	100.0%
42. Provide personal protective equipment for use by employees	290	68.9%
43. Establish an isolation area for confirmed cases of infection due to COVID-19, with restrictions on the movement of people and employees	16	3.8%
44. Thoroughly clean and disinfect rooms and furniture surfaces	286	67.9%
45. Pack and transport linen in plastic bags to avoid direct contact	177	42.0%
46. In F&B areas, maintain a minimum distance between tables (2 m) and chairs (1 m)	246	58.4%
47. Install acrylic/glass protection in the employee cafeteria, in the case of a buffet	82	19.5%
48. Have one person serve other employees at the cafeteria buffet.	63	15.0%
49. Maintain a schedule for use in the employee cafeteria to avoid crowds	368	87.4%
50. In the employee cafeteria, individual meals must be served sealed	77	18.3%
51. Cover trays and food when transporting it to the room, in room service	390	92.6%
52. Respect the minimum distance between tables (2 m) and chairs (1 m) in event areas	119	28.3%
53. Other	0	0.0%

According to which of the HSP are still followed by the hotel today, of the 52 items the following were the most marked: "use alcohol gel to disinfect hands" and "maintain physical distance" as all participants marked these items. The least marked items were "checking guests’ temperature when checking in at the hotel" and "establish an isolation area for confirmed cases of COVID-19 infection, with restriction of movement of people and employees," with no marks.

In the question, about the use of "HSP" in their personal life, of the 17 items, the most marked were "maintain the use of hand sanitizer" and "remove shoes when entering their house," both with 72.9% (307 people), followed by "keeps a bottle of alcohol gel with their personal belongings," with 65.1% (274 people). The following were the least marked: "working from home," with 1.9% (8 people); "wear masks correctly at work," with 3.6% (15 people), and "have mats with disinfectants," with 6.7% (28 people) (Tables 3 and 4).

**Table 3:** Hygiene and safety protocol items maintained at the hotels after the COVID-19 pandemic (421 answers)

Variable	n	%
1. Wear masks correctly at work	396	94.1%
2. Use alcohol gel to disinfect hands	421	**100.0%**
3. Maintain physical distancing	421	**100.0%**
4. Have acrylic barriers at the reception	334	79.3%
5. Keep employees belonging to risk groups away from work	70	16.6%
6. Reduce the number of employees entering the rooms (cleaning and/or room service)	383	91.0%
7. Pack bed linen and towels	353	83.8%
8. Pack plates, glasses, and cutlery	366	86.9%
9. Reduce the number of passengers in elevators	381	90.5%
10. Have mats with disinfectants	401	95.2%
11. Work from home	36	8.6%
12. Check employees’ temperature upon arrival at work	317	75.3%
13. Remove self-service coffee and/or other drinks from social areas (thermal/machine)	334	79.3%
14. Have alcohol gel dispensers in elevators	371	88.1%
15. Remove newspapers and magazines from lobbies/reception	355	84.3%
16. Use soap and disposable towels (washbasins) in addition to activated trash cans without using their hands.	407	**96.7%**
17. Conduct campaigns, via online communication channels, about the hotel policy on safety protocols	409	**97.1%**
18. Reduce the amount of furniture in social areas	211	50.1%
19. Clean rooms with ventilated areas	371	88.1%
20. Perform wet cleaning instead of dry cleaning and use of a vacuum cleaner	347	82.4%
21. Make reservations for customers in restaurants and bars with reduced spaces	128	30.4%
22. Keep employees to serve guests at buffets, which must be completely closed	259	61.5%
23. Install acrylic/glass protection at the buffet in restaurants	226	53.7%
24. Do not perform table mise en place to avoid cross-contamination	243	57.7%
25. Provide and maintain good ventilation in social areas and back offices	197	46.8%
26. Disinfect leisure areas (gyms, saunas, solariums, etc.) after use and only with reservations	142	33.7%
27. Disinfect air conditioning weekly	310	73.6%
28. Cover payment machines with plastic film	408	**96.9%**
29. Encourage contactless payments	345	81.9%
30. Disinfect luggage upon check-in	19	4.5%
31. Cover TV and air conditioning remote controls with plastic film	70	16.6%
32. Have signs with COVID-19 prevention guidelines	199	47.3%
33. Disinfect TVs weekly	185	43.9%
34. Cover power cables with plastic film	7	1.7%
35. Use disposable amenities as a priority	312	74.1%
36. Disinfect room key cards (check-in)	381	90.5%
37. Remove trash more frequently	326	77.4%
38. Check guests’ temperature upon checking in at the hotel	0	0.0%
39. Disinfect the key card after the guest returns it (check-out)	346	82.2%
40. Have alcohol gel dispensers in social areas	404	96.0%
41. Have alcohol gel dispensers in internal areas	404	96.0%
42. Provide personal protective equipment for use by employees	144	34.2%
43. Establish an isolation area for confirmed cases of infection due to COVID-19, with restrictions on the movement of people and employees	0	0.0%
44. Thoroughly clean and disinfect rooms and furniture surfaces	156	37.1%
45. Pack and transport linen in plastic bags to avoid direct contact	119	28.3%
46. In F&B areas, maintain a minimum distance between tables (2 m) and chairs (1 m)	43	10.2%
47. Install acrylic/glass protection in the employee cafeteria in the case of a buffet	23	5.5%
48. Have one person serve other employees at the cafeteria buffet.	8	1.9%
49. Maintain a schedule for use in the employee cafeteria to avoid crowds	326	77.4%
50. In the employee cafeteria, individual meals must be served sealed	39	9.3%
51. Cover trays and food when transporting it to rooms in room service	355	84.3%
52. Respect the minimum distance between tables (2 m) and chairs (1 m) in event areas	63	15.0%
53. Other	0	0.0%

**Table 4:** Hygiene and safety measures adopted in personal life after the pandemic (421 answers)

Variables	n	%
1. Wear masks correctly at work	15	**3.6%**
2. Wear masks correctly when "outside"	65	15.4%
3. Maintain physical distancing	181	43.0%
4. Avoid crowded elevators	147	34.9%
5. Work from home	8	**1.9%**
6. Prefer contactless payment	234	55.6%
7. Remove trash more frequently	218	52.0%
8. Cover remote controls for electronic devices with PVC film	76	18.1%
9. Keep alcohol gel in use	307	**72.9%**
10. Keep a bottle of alcohol with their personal belongings	274	**65.1%**
11. Disinfect electronic devices weekly	62	14.7%
12. Have disinfectant mats	28	6.7%
13. Use alcohol gel to disinfect their hands	206	48.9%
14. Always keep their home well ventilated	41	9.7%
15. Take off their shoes when they enter their house	307	**72.9%**
16. Keep the habit of washing their hands constantly throughout the day	74	17.6%
17. Disinfect their groceries when they get home	0	**0.0%**

Regarding the association between the amount of training and the hotel standard, of the 421 respondents, Cluster 1 comprises 38.5% (162 responses) and Cluster 2, 61.5% (259 responses). The frequency of training was significantly higher in hotels belonging to Cluster 2 than the ones in Cluster 1 (χ^2^ for linear trend = 53.05 p < 0.0000001).

Regarding the association between participants’ gender and the adoption of safety and hygiene measures, the answer yes was significantly higher in female respondents to the items "carrying a bottle of hand sanitizer with your belongings," "frequently cleaning electronic devices," and "disinfecting hands with alcohol gel" (Table 5).

**Table 5:** Association between gender of the participant and selected variables

Variable		n (%)	p-value
**Carrying a bottle of hand sanitizer**			
	Female	105 (48.6)	0.0001
	Male	42 (20.5)	
**Frequently cleaning electronic devices**			
	Female	47 (21.8)	0.0001
	Male	15 (7.3)	
**Disinfecting hands with alcohol gel**			
	Female	175 (81.0)	0.0001
	Male	31 (12.4)	

As for the association between the department of the hotel in which the respondents worked and the number of times they had COVID-19, the participants in a management/financial department had COVID-19 0.56 times on average; the ones from a lodging department, 0.87 times; and the ones from a food/beverage department, 0.94 times. The analysis of variance showed a significant difference between these groups (z = 3.23; p-value = 0.041).

Participants’ educational level, their age group, and the number of COVID-19 vaccine doses they had received showed an association. The ones with a higher educational level and older ones received a significantly lower number of vaccine doses (Table 6).

**Table 6:** Associations between the number of COVID-19 vaccine doses and selected variables

Variable		n	Mean number of doses	p-value
**Education**				
	Elementary	48	3.31	0.029
	High school	77	3.73	
	Undergraduate	265	3.41	
	Graduate studies	31	2.87	
**Age group**				
	18 – 40	377	3.54	0.001
	41 and older	44	2.43	

## DISCUSSION

Only a small fraction of the articles in our literature review studied the pandemic using the view of employees in the hospitality sector. Most sought to know the view of managers rather than that of employees with lower hierarchical positions, such as workers in the operational and supervisory areas^29-35^. No analyzed article either directly questioned the hygiene and safety protocols in hotels during the pandemic or were carried out in Brazil or Latin America.

A survey with 104 hotel employees in the city of Kigali, Rwanda, in July 2020, aimed to assess basic knowledge and attitudes about COVID-19^28^. Its results showed that, in general, employees had satisfactory knowledge and attitudes regarding appropriate practices to prevent the transmission of COVID-19. Even so, the author states that educational interventions are necessary for better prevention.

The article carried out in 2020^29^ included 823 Spanish hotel managers. It sought to evaluate the actions taken to prevent the spread of the virus in these hotels and reached five conclusions/recommendations: 1) Testing customers and employees should be fast and accessible throughout the European Union; 2) Training should be carried out by public authorities and the private sector to assemble experienced teams committed to clients’ health and safety; 3) The HSP established by the public authorities should be observed and adjusted gradually in hotels and in tourist arrivals to the country; 4) Practices require periodic updates; and 5) hotels must maintain constant "surveillance" to ensure their customers’ safety.

The data presented in the article aimed to determine the levels of knowledge^31^, beliefs, and practices about infectious diseases in 128 employees, including the environmental quality of hotels in Wuhan, China. The results showed moderate levels of knowledge and beliefs and good health practices. However, regarding the environment, quality was negatively correlated with the classification of the hotels, that is, the lower their classification, the worse their environment. Thus, hotels should expand employee training and improve the hygiene quality of hotel environments.

These data^28-30^ resemble ours regarding the association between "amount of training" and "hotel standard." Our results clearly show that one- and two-star hotels and unclassified ones (cluster 1) preferably carried out "bimonthly," "quarterly," or " no" training. Among the interviewees who stated that having received no training, the percentages of this group of hotels were 4.5 times higher than those of higher standard hotels, which shows that these hotels should focus on employee training and on improving the hygiene of their establishments^28^.

On the other hand, the hotels in Cluster 2, according to the interviewees, carried out training preferably "weekly," "biweekly," and "monthly". This group of hotels carried out weekly training in a proportion 10 times that of the hotels of a lower standard. The results of Cluster 2 are important to confirm the need for educational interventions to improve employees’ knowledge and attitudes and prevention^28^.

Our results were also related to the ones in an article^29^, especially with the conclusions "training should be carried out by public authorities and the private sector to assemble experienced teams committed to the health and safety of the client," as, even to a varying degree, 94.8% of the interviewees stated having received some type of training on the HSP of COVID-19 and that "hotels needed to maintain constant "vigilance" to ensure the safety of their customers" as 222 respondents reported that training occurred more frequently ("weekly," "biweekly," and "monthly"), that is, almost 57% of employees received some training during the COVID-19 pandemic.

The article developed in 2022^31^ describes the practices adopted by the health system of New South Wales, Australia. It makes clear the importance of the rules defined by the bodies responsible for local health in the control of the pandemic. Although 5.2% of the interviewees stated that having received no training on "HSP" in their work and in their daily lives, these interviewees indicated several HSP that they remembered existing in the hotels in which they worked.

An article^32^ analyzed COVID-19 tests from 679 employees from 21 hotels and restaurants from July to December 2020. However, when analyzing an outbreak in the region of Lübeck, Germany, in October 2020, it found that the group with the highest number of infected employees belonged to the "cleaning" sector, which was a risk factor for potential infection. These data corroborate the data in our study, which showed a higher frequency of the disease in employees in hotel operating areas, possibly because these employees had the most direct contact with guests, wander more in the means of accommodation, and maintain direct contact with other employees in the other operational areas of the company.

An article^33^ sought to know what HSP-related measures hotel guests demanded the most. It concluded that women had a greater demand than men on a set of measures that would ensure good hygiene conditions. Another survey^34^ questioned employees who received guests who had to carry out the mandatory 14-day quarantine about their knowledge, practices, and attitudes about the pandemic. Its results show that 62.41% had good knowledge and 94.7% had a positive attitude toward COVID-19 but only 78.2% had good practices. These employees also took personal care, such as avoiding crowds (80.6%) and wearing face masks (97.6%).

These results^33,34^ are in line with our study, which shows that female employees are more concerned with maintaining HSP in their personal lives than male ones. The data from our survey draws attention to the fact that women "disinfect their hands with alcohol gel" in a proportion 5.5 times higher than men.

As for the result on the age groups and the number of doses of the vaccine employees had received, the group aged up to 40 years had received an average of 3.54 doses, whereas those aged over 40 years received an average of 2.43 doses. However, other studies — carried out in Hong Kong^35^, Egypt^36^, a compilation of data from 20 countries^37^ and Turkey^38^ — have conflicting data on vaccine acceptance across age groups. The studies agree that to increase the acceptance of the vaccine, actions should have been taken to combat distrust in health authorities, to act against low confidence in the vaccine, to explain the misconceptions of the vaccine urged via fake news, and making clear the side effects of vaccines.

The hotel industry in Sao Paulo should also have participated decisively in this explanation effort as part of the organizational culture^39^ of hospitality companies should be the development of a positive work environment conducive to knowledge and personal and professional growth, and which should have provided as one of its focuses reliable information about vaccination at the time.

As in Hong Kong^35^ and Turkey^38^, Brazil faced problems with vaccination, with lies about possible side effects (including conflicting information from official government bodies) that ended in ideological political issues that contaminated a discussion that should be based on scientific data. In our study the age group with the greatest rejection of the use of vaccines was the oldest (aged over 60 years), evincing the past need for a different communication on the behalf of the official health agencies and the Brazilian authorities. A way to combat misinformation would be to create a communication strategy with objective data that effectively conveyed information and respected the local culture^38^. When analyzing the incorporation of information technology by hotels in Spain as part of the response to the pandemic, Palacios-Marqués *et al*.^40^ point to a causal relationship between technological incorporation and learning in the hotel industry and recommend that the industry develop an IT-based structure as a way to expand its learning capacity and improve the services provided.

The main limitations of this study are related to the time interval between the most intense period of the pandemic (2020-2022) and the fieldwork (2023-2024). Information on the HSP used in the hotels, the amount of training, the time worked in the hotel, etc. may have undergone losses due to interviewees’ memory. Other limitations include the fact that this study was conducted in a non-randomly selected sample and the fact that survey was carried out online with closed answers, which may have made it difficult for the interviewees to express themselves more clearly. On the other hand, the fact that it was an interview with closed answers made it easier for the interviewees to be freer in the choice of answers.

## CONCLUSION

This study found a direct relationship between the quality of the surveyed hotels and the frequency of training received by employees. The hotels in Cluster 2, which included the three-, four-, and five-star hotels, that is, the highest standard, offered training more frequently than Cluster I (unclassified and one - and two-star hotels).

Among the possible reasons for that, we may infer that hotels with lower quality standards tend to have fewer resources toward operational training with employees, which would involve training on HSP; they usually have no human resources sectors, which distances them from the daily life of the hotel and their immediate needs; perhaps due to lack of interest from management in verifying the use and maintenance of the HSP.

This study found a high adherence to vaccination against COVID-19 but lower at the higher educational levels. The possible reasons for that include fake news on the vaccine that emerged throughout the pandemic and political issues that last to the present day.

Hotel employees scarcely maintained HSP in their daily lives. However, the results of this survey made it clear that women maintained a greater concern with their protection against diseases than men. Women "disinfected their hands with alcohol gel" in a proportion 5.5 times higher than among men. This result corroborates the data of two studies abroad, which also showed a greater concern in women toward maintaining HSP in their daily lives.

In general, this study shows that people and companies learned a few lessons from the pandemic. The respondents made it clear that few still follow the hygiene issues that were common during the pandemic in their daily lives, such as washing their hands frequently, avoiding crowds, constantly using alcohol gel to disinfect their hands, cleaning electronic devices, etc.

Those who still follow some measures to combat the pandemic do so because they realize that some of the measures implemented during the fight against COVID-19 would be important for health, prevention of other diseases, and that adopting them as daily habits would take neither much time nor money. Regarding the companies, employees stated that that they almost immediately abandoned the introduced HSP introduced as soon as the pandemic cooled down. Reception services no longer have acrylic barriers, housing units no longer prioritize wet cleaning, restaurants no longer impose a minimum safety distance between customers, dispensers at receptions no longer contain hand sanitizer, etc.

Hotel companies and employees should retain the lessons from HSP during the pandemic as they may be necessary soon. Thus, the more we remember how the COVID-19 pandemic was faced, the lower the chances of a similar and/or new disease contaminating employees in the hotel industries.

## Data Availability

The complete anonymized dataset supporting the findings of this study is available from https://sites.usp.br/epi-di/
